# Food addiction and gender aspects: Risk factors, co-morbidites and treatment

**DOI:** 10.1192/j.eurpsy.2023.136

**Published:** 2023-07-19

**Authors:** E. Sönmez Güngör

**Affiliations:** Psychiatry, Erenköy Mental Health and Neurological Diseases Training and Research Hospital, Istanbul, Türkiye

## Abstract

**Abstract:**

Food addiction (FA) is characterized by poorly controlled intake of highly-palatable, calorically-dense, high-sugar, high-fat, and usually processed foods. Although not existing in the DSM-5 or ICD, it has been a widely discussed clinical and research entity for more than a decade, in the understanding of both obesity and also disordered eating behaviours. Most studies indicate that risk factors for FA are similar to substance use disorders. FA is also found to be linked with emotion regulation problems, impulsivity, distinct personality features, and other major psychopathologies including depression and anxiety. Morevoer, in several studies, female gender is found to be associated with more prevalent food addiction, up to 7 times. Female gender is also a predictor of severe food addiction, and high reward sensitivity was significantly associated with more severe FA symptoms in females. It is known that women are more likely to self-medicate than men in the acquisition phase of addiction and show a more rapid escalation of use than men (telescoping), whereas in general substance use disorders are much more common in men. The wide availability of hyperpalatable, high-calorie, and inexpensive food and the contextual and social factors that do not necessarily prevent women from consuming these drugs, contrary to that of illicit drugs, might explain why FA is more frequently observed in women than in men. Also, biological factors, hormonal changes and body-image disturbances might also be among the reasons why females are more affected from FA. Aspects related to gender should be taken into account for proper recognition and treatment of FA, a diagnosis of growing importance.

**Disclosure of Interest:**

None Declared

